# Postpartum Family Planning Use and Its Determinants among Women of the Reproductive Age Group in Low-Income Countries of Sub-Saharan Africa: A Systematic Review and Meta-Analysis

**DOI:** 10.1155/2021/5580490

**Published:** 2021-08-20

**Authors:** Tesfalem Tilahun Yemane, Getahun Gebre Bogale, Gudina Egata, Tilahun Kassa Tefera

**Affiliations:** ^1^Department of Nursing, Dessie Health Science College, Dessie, Ethiopia; ^2^Department of Health Informatics, School of Public Health, College of Medicine and Health Sciences, Wollo University, Dessie, Ethiopia; ^3^Department of Public Health Nutrition & Dietetics, School of Public Health, College of Health Sciences, Addis Ababa University, Ethiopia

## Abstract

**Background:**

Postpartum family planning is the initiation and use of family planning services within the first 12 months following childbirth. Postpartum contraceptives reduce maternal and infant mortality by preventing unplanned and unwanted pregnancies and by spacing pregnancies at least two years after the previous birth. Thus, it is usually designed as an integral part of reproductive and maternal and child health programs. Therefore, the aim of this systematic review and meta-analysis is to estimate the pooled prevalence of postpartum modern contraceptive use and identify its determinants in low-income countries of sub-Saharan Africa.

**Methods:**

A systematic review and meta-analysis of published and unpublished studies were used. PubMed, HINARI, ScienceDirect, Cochrane Library, Wiley Library, ETH Library, and Google Scholar were used to search all articles. STATA 14 software was used for data analysis. Funnel plots and Egger's test were used to examine the risk of publication bias. Heterogeneity was checked by using Cochran's *Q* test and *I*^2^ test. A random effect model was computed to estimate the pooled prevalence.

**Results:**

A total of 33 articles were included. The pooled prevalence of postpartum contraceptive use in low-income countries of sub-Saharan Africa was 37.41%, 95% CI: (31.35, 43.48%). Secondary and above level of education (AOR 2.09, 95% CI: (1.52, 2.86)), discussion with husband (AOR 3.68, 95% CI: (1.96, 6.89)), resumption of menses (AOR: 3.98, 95% CI: (2.62, 6.03)), ANC follow-up (AOR; 5.10, 95% CI: (3.57, 7.29)), knowledge of modern family planning (AOR: 5.65, 95% CI: 3.58, 8.93)), and family planning counseling during ANC (AOR =5.92, 95% CI: (2.54, 13.79)) were found to be determinants of postpartum contraceptive utilization.

**Conclusion:**

In this systematic review and meta-analysis, the prevalence of postpartum modern contraceptive use was found to be low compared to the existing global recommendations. Therefore, empowering maternal education, delivering adequate counseling, and strengthening existing integrated maternal and child health services are highly recommended to increase postpartum contraceptive use. This trial is registered with CRD42020160612.

## 1. Introduction

Postpartum family planning (PPFP) is the initiation and use of family planning services within the first 12 months after childbirth to prevent unintended and closely spaced pregnancies [1, 2]. The postpartum period is critical for addressing high unmet needs in family planning and is used for reducing the risk of closely spaced pregnancies [3]. According to the World Health Organization (WHO), it recommends that interpregnancy intervals should be at least 2 years [4]. Short birth intervals increase the risk for the health of both the mother and the child, such as risk of preterm birth, low birth weight and small for gestational age, increased chances of chronic undernourishment, stunted growth, and child mortality [5, 6]. Using family planning during the postpartum period may help women to space births by at least 24 months, and this can also help to reduce maternal and child mortalities by 30% and 10%, respectively [7]. When a pregnancy occurs less than six months after a previous delivery, the risk of low birth weight and prematurity doubles, and children born less than 24 months after a previous birth are 60% more likely to die during infancy than those born more than 24 months [8].

Postpartum contraceptive utilization remains low in sub-Saharan Africa [9]. Since the uptake of PPFP was low, the significant factors influencing the uptake of PPFP are the level of education, perinatal family planning, counselling, menses return, breastfeeding status, return of sexual activity, fear of side effects, and low perceived risk of getting pregnant [10].

Maternal health remains a major global concern since pregnancy and childbirth are the leading causes of morbidity, mortality, and disability among women of the reproductive age group [11]. Globally, more than 9 out of 10 women want to avoid pregnancy for 2 years after childbirth [2]. According to DHS data from 21 low- and middle-income countries between 2005 and 2012, almost all (95%) women 0-12 months postpartum wanted to avoid pregnancy in the next 24 months, but less than one-third (31%) were using any method of contraception. Sixty-one percent (61%) of postpartum women had an unmet need for family planning [3, 12].

According to studies done in five low-income countries, the rates of postpartum modern contraceptive usage varied widely, and the unmet need ranged from 25% to 96%. Fifty percent of women have an unmet need for family planning services among all women who wish to delay the future pregnancy [13]. It is also higher among women in developing regions, particularly in low-income countries of sub-Saharan Africa [14]. Even though the postpartum modern contraceptive is critical, studies suggest that its use varies widely across geographical regions of low- and middle-income countries [13]. Therefore, this systematic review and meta-analysis was aimed at estimating the pooled prevalence of modern family planning among postpartum women in low-income countries of sub-Saharan Africa.

## 2. Materials and Methods

### 2.1. Study Design and Protocol Registration

A systematic review and meta-analysis of published and unpublished studies were used. This systematic review and meta-analysis were carried out in accordance with the recommendation of the Preferred Reporting Items for Systematic Reviews and Meta-Analyses (PRISMA) 2015 statement [15]. The protocol of this systematic review and meta-analysis has been registered on the International Prospective Register of Systematic Reviews (PROSPERO), registration number CRD42020160612 available from https://www.crd.york.ac.uk/prospero/display_record.php?.

### 2.2. Criteria for Eligibility

#### 2.2.1. Inclusion Criteria

Articles were included in this systematic review and meta-analysis if they fulfilled all inclusion criteria:

- *Publication condition*: published studies, unpublished studies, and PhD dissertations which reported the outcome of interest were considered
- *Outcome of interest*: studies reported data on the prevalence of postpartum modern contraceptives or/and their determinants were considered
- *Publication year*: published articles between January1/2010 and January 1/2020 were included
- *Study design*: observational studies (cross-sectional)
- *Study setting*: all studies were conducted at the community or health institution level
- *Language*: articles published in English and French languages were included
- *Study area*: studies conducted in low-income countries of sub-Saharan Africa were included
- *Population*: articles on postpartum women were considered. Studies with data on contraceptive use in the first 12 months postpartum period were included. Studies that included follow-up data after an extended postpartum period were included if the data could be disaggregated by month, which only includes data during the first 12 months postpartum

#### 2.2.2. Exclusion Criteria

Articles which were not fully accessed after at least two email contacts of the primary author or failed to contact their primary authors were excluded.

### 2.3. Search Strategies

Relevant published studies were searched from the PubMed, HINARI, ScienceDirect, Wiley Library, ETH Library, and Cochrane Library electronic databases. Likewise, a search for grey literature was conducted using direct Google search and Google Scholar. Medical subject heading (MeSH), keywords, and thesaurus were used to identify selected PICO components. To combine search terms, Boolean operators (“OR,” “AND,” and “NOT”) were used. The following keywords were used in the search: “postpartum family planning” OR “planification familiale post-partum” OR “postpartum contraceptive” OR “contraceptif post-partum” AND Prevalence OR Epidemiology OR utilization OR Utilisation OR use AND determinants OR déterminante OR “Factors associated” OR “facteurs associés” OR predictors OR prédicteurs. This review included studies published in English and French between January 1, 2010, and January 30, 2020. The search was carried out from 01 January 2020 to 01 March 2020.

### 2.4. Study Selection

All articles identified for the review were imported to EndNote X7, and duplicated studies were excluded. All studies were initially examined for inclusion based on information contained in the titles alone and abstracts. Then, a full-text review was performed by two independent reviewers (TT and TK). Cohen's kappa agreement test was done to test interrater reliability. The kappa coefficient was *k* = 0.667 and *p* < 0.001 with an asymptotic standard error of 0.124, indicating the agreement between the two reviewers was substantial [16]. The PRISMA flow diagrams were used to summarize the selection procedure and process of the article [17].

### 2.5. Quality Appraisal

Two reviewers (TT and TK) independently assessed the risk of bias in the study. A modified version of the Newcastle-Ottawa Scale was used to appraise the quality of the studies [18]. The studies were divided into three categories: (0–4) low quality, (5–7) medium quality, and (8–10) high quality [19]. Any disagreement which arose between the two reviewers was solved through discussion and reached to consensus by involving a third reviewer. Those studies with medium (satisfying 50%) and high qualities were included for analysis.

### 2.6. Data Extraction

Data was extracted from all articles that met the inclusion criteria using Microsoft Excel spreadsheet. The data extraction tool was adopted from the Joanna Briggs Institute (JBI) data extraction checklist for observational studies [20]. The data extraction tool was pretested in 10 randomly selected studies. The extracted data was entered into a Microsoft Excel spreadsheet before being exported to the STATA 14 software. All relevant information was extracted by two independent reviewers (TT and TK), while the authors' name, study area, and journal were blinded. The discrepancies were solved through discussion. In the case of incomplete data on constructing two-by-two tables, the reported odds ratio with its confidence interval (OR; 95% CI) was used. Incomplete data was requested for by contacting the authors.

### 2.7. Data Analysis, Publication Bias, and Heterogeneity

STATA Version 14 (software) was used for data entry and analysis. Funnel plots and Egger's and Begg's tests were used to examine the possible risk of publication bias. Statistical heterogeneity was checked by using the Cochran's *Q* test and the *I*^2^ test, which shows the percentage of total variation across the studies due to heterogeneity rather than chance. The *I*^2^ statistics of below 25% are low heterogeneity, 25–50% is moderate heterogeneity, 51–75% is substantial heterogeneity, and above 75% is considerable heterogeneity [21]. A *p* value < 0.05 was used to declare heterogeneity. In the case of heterogeneity, the random effect (DerSimonian and Laird) model was used to estimate the pooled prevalence of postpartum modern contraceptive use. Forest plot and odds ratios with their 95% CI were used to present the pooled effect sizes. Subgroup analysis was conducted by region and study setting. A forest plot was constructed for each variable. Metaregression models were used to explore the relationship between the study-specific effect size and the study level covariates. Sensitivity analysis was used to measure the predictive power by excluding a single study.

## 3. Results and Discussion

### 3.1. Study Selection

A total of 743 articles were searched through the electronic databases: 301 articles from PubMed, 43 articles from HINARI, 122 articles from ScienceDirect, 51 from Cochrane Library, 219 from Google Scholar, 4 articles from Wiley Library, and again 4 articles from ETH Library. From these, 134 articles were excluded due to duplications, while the remaining 609 articles were reserved for further screening. Of these remaining articles, 320 and 241 articles were excluded by their titles and abstracts, respectively. A total of 48 full-text articles were assessed for eligibility criteria. Finally, 33 articles with appropriate quality were included in the final systematic review and meta-analysis. Furthermore, the PRISMA flow diagram was used to summarize the selection procedure (Additional file 1).

### 3.2. Characteristics of Original Studies

As described in Table 1, these 33 original articles were included in this systematic review and meta-analysis. The selected articles were published from 2010 to 2020. Two of them were unpublished articles [22, 23]. Regarding study design, all studies are cross-sectional in nature. The sample size of the included studies ranged from 248 (in Ethiopia, Eastern Africa) [10] to 3617 (in Malawi, Eastern Africa) [24]. In this study, 27,128 postpartum women were involved. Among the 33 included studies, most of the studies (87.88%) were from Eastern African countries. Out of 33 studies, 21 studies were conducted both in urban and rural settings [22–42], eight were in urban [10, 43–49], and four [50–53] were done in rural settings.

Among the 26 studies that reported the mean age of postpartum women, it ranged from 25 [31] to 30.8 years [33]. The highest and lowest prevalences of postpartum modern contraceptive use (80.3% and 3.41%, respectively) were reported from Addis Ababa, Ethiopia [44], and Rural Guinea, West Africa [51], respectively. The response rate of included studies ranges from 94.3% [51] to 100% [10, 23, 30, 38, 45, 50]. Finally, the quality score of the included studies ranges from 6 up to 9 out of 10 points. Further descriptions and characteristics of the studies selected for this systematic review and meta-analysis are presented in Table 1.

### 3.3. Quality of Included Studies in the Systematic Review

Modified versions of the Newcastle-Ottawa Scale [18] were used to assess the quality of the selected articles. Quality scores were defined based on the presence of sample representativeness, sample size, reporting of response rate, ascertainment of study outcomes, control of confounder, and quality of descriptive statistics reporting. Regarding the quality score of the included studies, 16 of the 33 studies had high quality (8-10 points) and the remaining 17 studies were medium-level quality (5-7 points).

### 3.4. Heterogeneity and Publication Bias

**Figure 1:**
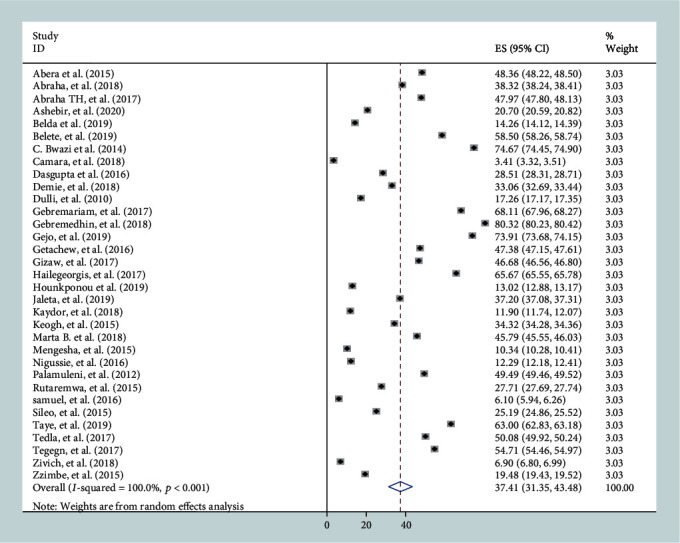
Forest plot of the pooled prevalence of postpartum modern contraceptive use with its 95% confidence interval among women of the reproductive age (15-49 years) group in their first 12 months after delivery in low-income countries of sub-Saharan Africa, 2020.

**Figure 2:**
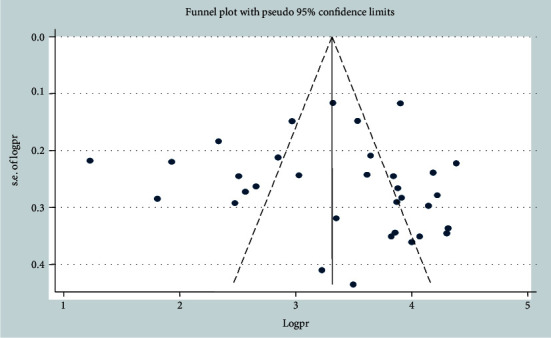
Funnel plot to check publication bias of the 33 included studies in low-income countries of sub-Saharan Africa, 2020.

Statistical heterogeneity was checked by using Cochran's *Q* test and the *I*^2^ test. In this analysis, considerable heterogeneity was observed across the included studies and detected by the Cochran *Q* test (*Q* test *p* < 0.001) and *I*^2^ statistics (*I*^2^ = 100%) (Figure 1). Therefore, DerSimonian and Laird's random effect model was used. Publication bias was checked by funnel plots. The shape of the funnel plots indicates a slightly asymmetrical distribution (Figure 2). Moreover, to ascertain the funnel plot, Begg's and Egger's objective tests were conducted. Begg's and Egger's test results showed that there was no statistically significant publication bias across the included studies (*p* = 0.69 and *p* = 0.50, respectively).

### 3.5. Meta-Analysis

In this meta-analysis, considerable heterogeneity (*I*^2^ = 100%, *p* < 0.001) was observed across the studies. Therefore, a random effect meta-analysis model was used to estimate the pooled effect of postpartum contraceptive use. As shown in the forest plot, the results of 33 included studies indicated that the pooled prevalence of postpartum contraceptive use in low-income countries of sub-Saharan Africa was 37.41%, 95% CI: (31.35, 43.48%) (Figure 1).

### 3.6. Subgroup Analysis

Subgroup analysis was performed based on the regions where the studies were conducted, sample size, study setting, and year of publication of the studies to assess possible causes of considerable heterogeneity. Based on subgroup analysis, Eastern Africa had the highest prevalence of postpartum contraceptive use (41.36%, 95% CI: (35.2, 47.52)), followed by Western Africa (9.45%, 95% CI: (2.69, 16.2)) and others (central Africa 6.9%). Regarding the study setting, the prevalence of postpartum contraceptive utilization was 40.97% (95% CI: (33.38, 48.55)) among studies conducted at the community level (Table 2). A prevalence of (38.66%, 95% CI: (28.4, 48.9)) was observed in studies which have been published since 2016 (Table 2). However, the results of the subgroup analysis indicated that the source of considerable heterogeneity was not because of the study regions, sample size, study setting, and year of publication of the studies.

### 3.7. Determinants of Postpartum Modern Contraceptive Use in Low-Income Countries of Sub-Saharan Africa

**Figure 3:**
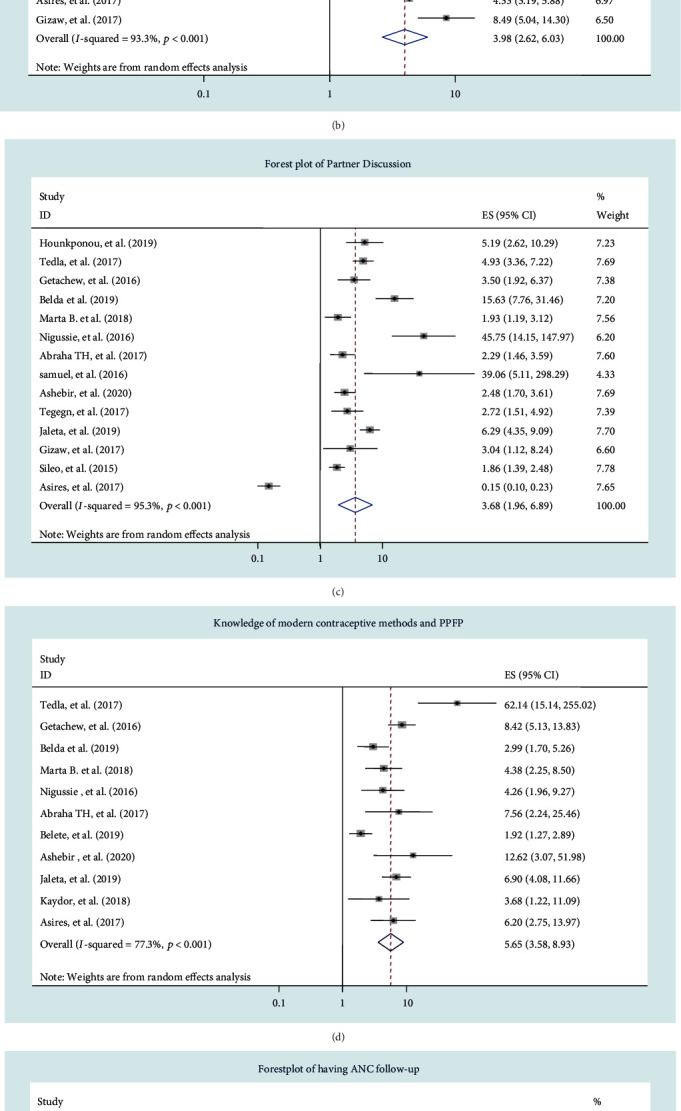
(a–f) Forest plot displaying the pooled odds ratio of the associations between the use of PPFP and its determinants: (a) mothers' educational status, (b) resumption of menses, (c) having partner discussion, (d) having knowledge of modern contraceptive methods, (e) having ANC follow-up, and (f) family planning counselling during ANC, in low-income countries of sub-Saharan Africa, 2020.

Of the 33 studies identified, 31 studies were included to identify determinants of postpartum modern contraceptives. The two studies [24, 31] were excluded because of insufficient data to extract two-by-two tables, and the reported odds ratio does not have a confidence interval. Metaregression was performed for all selected determinants to determine the possible sources of variation, but there was no statistical significance. As a result, to determine the associations, a random effect model was computed (Figures 3(a)–3(f)).

#### 3.7.1. Association between a Mother's Educational Status and Postpartum Modern Contraceptive Use

To determine the association of a mother's educational status with postpartum modern contraceptive use, 18 studies were included. The findings of the studies indicated that those mothers who had secondary and above level of education were 2.09 times more likely to use postpartum contraceptives (AOR 2.09, 95% CI: (1.52, 2.86)), compared to those who had primary education and below.

In this analysis, the test statistics indicated that considerable heterogeneity (*I*^2^ = 89.7% and *p* = 0.000) was presented across the included studies. To explore this heterogeneity, a sensitivity analysis was done and there was no significant change in the overall results of OR. In addition, Begg's and Egger's tests revealed the absence of statistically significant publication bias (*p* = 0.058 and *p* = 0.664, respectively).

#### 3.7.2. Resumption of Menses and Use of Postpartum Modern Contraceptives

A total of 15 studies [22, 25–30, 34, 42–46, 48, 49] were included. The findings revealed that women who experienced menstruation again after giving birth were four times more likely to use postpartum contraception than mothers who experienced amenorrhea in the postpartum period (AOR: 3.98, 95% CI: (2.62, 6.03)). Considerable heterogeneity was detected (*I*^2^ = 93.3%; *p* = 0.000) (Figure 3(b)). The results of sensitivity analysis indicate that there is no significant change in the overall results of OR. The results of Begg's and Egger's tests showed that there was no statistically significant publication bias across 15 studies (*p* = 0.69 and *p* = 0.50, respectively).

#### 3.7.3. Partner Discussion and Use of Postpartum Modern Contraceptives

The analysis included 14 studies [22, 26–28, 30, 34, 35, 39, 41, 43, 46, 47, 49, 53]. Studies revealed that discussing with the partner was significantly associated with the use of PPFP (AOR 3.68, 95% CI: (1.96, 6.89)) (Figure 3(c)). The selected studies exhibited considerable heterogeneity (*I*^2^ = 95.3% and *p* < 0.001). As a result, a random effect meta-analysis was employed. Publication bias was checked by using Begg's test (*p* = 0.125) and Egger's test (*p* = 0.213) which revealed that there was no evidence of publication bias.

#### 3.7.4. Having Knowledge of Modern Contraceptive Methods

From the meta-analysis of eleven [22, 26–30, 35, 36, 39, 43, 49] studies, knowledge of modern contraceptive methods was significantly associated with the use of PPFP (AOR: 5.65, 95% CI: 3.58, 8.93)). The overall heterogeneity was *I*^2^ = 77.3% and *p* < 0.001. As a result, a random effect meta-analysis was used. Publication bias assessed by using Begg's and Egger's tests revealed that there was a low possibility of publication bias with *p* values of 0.28 and 0.08, respectively.

#### 3.7.5. Having ANC Follow-Up

From the results of nine included studies [22, 25, 35, 39, 41, 43, 45, 48, 49], the heterogeneity test showed the presence of substantial heterogeneity (*I*^2^ = 52.8%). The pooled effect sizes of PPFP utilization among women who have attended at least one ANC visit were five times more likely (AOR; 5.10, 95% CI: (3.57, 7.29)) to use PPFP compared to those women not having ANC visits (Figure 3(e)). Begg's and Egger's tests for publication bias showed no statistical evidence of publication bias (*p* = 0.75 and *p* = 0.899, respectively).

#### 3.7.6. Family Planning Counselling and the Use of Postpartum Modern Contraceptives

Women who received family planning counselling during antenatal care were nearly six times more likely to use modern contraceptives in the postpartum period than those who did not (AOR =5.92, 95% CI: (2.54, 13.79)). The heterogeneity test showed that considerable heterogeneity was found (*I*^2^ = 93.2%, *p* = 0.000) between the studies (Figure 3(f)). To reduce the random variation, a sensitivity analysis was done, but did not bring a significant change in the overall results of OR.

### 3.8. Discussion

In this systematic review and meta-analysis, the overall pooled prevalence of postpartum modern contraceptive use in low-income countries of SSA was 37.41%. However, use varied regionally, with the highest prevalence of postpartum contraceptive use observed in Eastern Africa (41.36%), followed by Western Africa (9.45%) and Central Africa (6.9%). This finding is in line with a meta-analysis conducted in low- and middle-income countries, which was found to be 41.2% [54]. However, the current finding is lower than studies done in lower middle-income countries of South Asia (Bangladesh, 53%) [55], but higher than studies done in India (23%, 25.4%) [56, 57] and Pakistan (24.6%) [58]. The possible explanations for this variation might be due to heterogeneous socioeconomic, sociodemographic, and cultural differences between the populations.

In our meta-analysis, higher educated mothers were more likely to use PPFP than mothers with a low education level. It is in line with studies conducted in low- and middle-income countries [7, 54]. The possible explanations might be that when women's educational status increases, they will have better health care seeking behavior, they understand the benefits and disadvantages of contraceptives, and they will have the right information about fertility and contraception. Therefore, empowering maternal education helps them to make an informed decision on their fertility and for better maternal and child health.

Women who resumed menstruation after giving birth were more likely to use PPFP than mothers who experienced postpartum amenorrhea. This finding is comparable with the results of a meta-analysis conducted on PPFP [54, 55]. This is explained by the return of menstruation after delivery, which leads women to believe that once menstruation returns, the likelihood of becoming pregnant increases. Thus, they are more likely to start using a contraceptive. However, many women do not begin using a contraceptive until menstruation has resumed. Therefore, it is required to educate women; biologically, a woman may ovulate before the first menstruation has returned following childbirth.

Discussing with partners about PPFP was significantly associated with its use. Women who had a discussion with partners were more likely to use PPFP than their counterparts. The current finding is supported by a meta-analysis conducted in low- and middle-income countries [54]. This suggests that male involvement and support with family planning helps women to adopt more convenient methods with confidence.

In this finding, women who had knowledge of PPFP were more likely to use PPFP than those who did not. It is in line with the findings of a meta-analysis [54], but studies conducted in Malawi [31] showed that having knowledge of PPFP services is not significantly associated with the use of PPFP. Therefore, the pooled effect of this finding suggests having knowledge about modern contraceptives is a significant input to adopting family planning services. Therefore, creating awareness and promoting knowledge relating to modern contraceptives is required.

Women who had at least one ANC visit during pregnancy were more likely to use postpartum contraception than women who did not have an ANC visit. In contrast to the findings of the other included studies, ANC utilization was not statistically significant with the use of PPFP in a study conducted in North Gondar, Ethiopia [38]. This finding is in line with USAID findings from 17 countries and USAID DHS Comparative Reports [6, 55]. The possible explanation for this finding might be that women who used ANC had more exposure to information on birth spacing and complications of the short birth interval for both the mother and newborn.

Women who received proper family planning counselling during antenatal care were more likely to utilize postpartum contraceptives. However, the findings of a study conducted in South Africa [59] contradict this; it shows that contraceptive counselling during antenatal visits could have no impact on contraceptive use, whereas the current finding is in line with a meta-analysis conducted in low- and middle-income countries [54]. This might be due to frequent and proper counselling provided by health care providers about contraceptive use and the risk of closely spaced pregnancies. Focused ANC incorporates ANC counselling sessions. Due to this, the ANC providers also give more attention to family planning counselling; this may be another possible justification for this finding.

### 3.9. Strengths and Limitations

We used extensive and comprehensive search strategies systematically from multiple databases. Published and unpublished studies and grey literature were included and evaluated for methodological quality using a standardized tool. However, this review represented three regions of SSA, but the majority of studies were obtained from the East Africa region. Therefore, the results may not be strongly representative for the other regions due to the small number of studies included.

## 4. Conclusions

Postpartum contraceptive utilization is low and not optimal compared to the global recommendation on postpartum family planning. A global increase in postpartum contraceptive use can help in reducing maternal and child mortality and improving the lives of women and their families. Secondary and higher education levels, resumption of menstruation, discussion with husbands about the use of PPFP, knowledge of modern contraceptive methods, and use of maternal health services (ANC follow-up and family planning counseling during ANC) were all significantly associated with the use of modern postpartum contraceptives.

## Figures and Tables

**Table 1:** Descriptive summary of 33 studies included in the systematic review and meta-analysis of postpartum modern contraceptive use among women of reproductive age (15-49 years) group in their first 12 months after delivery in low-income countries of sub-Saharan Africa, 2020.

Author	Publication year	Country	Study design	Sample size	Women's mean age	Response rate (%)	Prevalence (95% CI)	Quality score (10 pts)
Abera et al. [25]	2015	Ethiopia	Cross-sectional	703	27.2	99.7	48.36 (48.22-48.5)	8
Abraha et al. [50]	2018	Ethiopia	Cross-sectional	1109	28.7	100	38.32 (38.24, 38.4)	7
Abraha et al. [26]	2017	Ethiopia	Cross-sectional	590	27.4	98.2	47.96 (47.8-48.13)	8
Ashebir and Tadesse [27]	2020	Ethiopia	Cross-sectional	681	30.26	99.3	20.7 (20.59-20.82)	8
Belda et al. [28]	2019	Ethiopia	Cross-sectional	505	27.67	98.0	14.26 (14.12, 14.93)	7
Belete et al. [29]	2019	Ethiopia	Cross-sectional	400	26.82	99.5	58.5 (58.26-58.74)	8
Bwazi et al. [31]	2014	Malawi	Cross-sectional	383	25	100.	74.67 (74.45-74.9)	6
Camara et al. [51]	2018	Guinea	Cross-sectional	381	25.2	94.3	3.41 (3.32-3.5)	6
Dasgupta et al. [52]	2016	Malawi	Cross-sectional	442	26.0	NR	28.5 (28.3-28.7)	6
Demie et al. [10]	2018	Ethiopia	Cross-sectional	248	27.40	100	33.06 (32.69-33.44)	7
Dulli et al. [32]	2010	Madagascar	Cross-sectional	840	26.8	100	17.26 (17.17-17.35)	6
Gebremariam and Gebremariam [33]	2017	Ethiopia	Cross-sectional	599	30.8	99	68.11 (67.96-68.27)	8
Gebremedhin et al. [44]	2018	Ethiopia	Cross-sectional	803	NR	94.6	80.32 (80.22-80.42)	8
Gejo et al. [45]	2019	Ethiopia	Cross-sectional	368	29.12	100	73.91 (73.68-74.15)	9
Getachew [22]	2016	Ethiopia	Cross-sectional	420	27.5	99.7	47.38 (47.15-47.61)	7
Gizaw et al. [34]	2017	Ethiopia	Cross-sectional	829	27.53	98.2	46.68 (46.56-46.80)	8
Asires et al. [43]	2017	Ethiopia	Cross-sectional	833	27.3	98.6	65.67 (65.55-65.78)	7
Hounkponou et al. [46]	2019	Benin	Cross-sectional	453	27.1	98.5	13.02 (12.9-13.17)	8
Jaleta et al. [35]	2019	Ethiopia	Cross-sectional	820	28	95.6	37.19 (37.08-37.31)	8
Kaydor et al. [36]	2018	Liberia	Cross-sectional	378	NR	100	11.90 (11.74-12.07)	7
Keogh et al. [37]	2015	Tanzania	Cross-sectional	2162	NR	NR	34.32 (34.28-34.36)	6
Berta et al. [30]	2018	Ethiopia	Cross-sectional	404	NR	100	45.79 (45.55-46.03)	8
Mengesha et al. [38]	2015	Ethiopia	Cross-sectional	899	28.3	100	10.34 (10.28-10.41)	8
Nigussie et al. [39]	2016	Ethiopia	Cross-sectional	545	31	98	12.29 (12.17-12.41)	8
Palamuleni [24]	2012	Malawi	Cross-sectional (DHS)	3617	NR	NR	49.49 (49.46-49.52)	7
Rutaremwa et al. [40]	2015	Uganda	Cross-sectional (DHS)	3298	29.7	100	27.71 (27.69-27.74)	7
Samuel [47]	2016	South Sudan	Cross-sectional	295	25.4	100	6.10 (5.94-6.26)	7
Sileo et al. [53]	2015	Uganda	Cross-sectional	258	25.85	100	25.19 (24.86-25.52)	7
Taye et al. [48]	2019	Ethiopia	Cross-sectional	546	27.57	97	63.0 (62.83-63.18)	8
Tedla [49]	2017	Ethiopia	Cross-sectional	623	27.5	98.7	50.08 (49.92-50.24)	8
Tegegn et al. [41]	2017	Ethiopia	Cross-sectional	382	28	99.7	54.71 (54.46-54.97)	8
Zivich et al. [42]	2018	DRC	Cross-sectional	522	NR	95.8	6.9 (6.80-70)	7
Zzimbe [23]	2015	Uganda	Cross-sectional	1792	NR	100	19.47 (19.43-19.52)	7

Note: DHS: demographic health survey; NR: not reported.

**Table 2:** Subgroup analysis for prevalence of postpartum modern contraceptive use among women of reproductive age (15-49 years) group in their first 12 months after delivery in low-income countries of sub-Saharan Africa, 2020 (*n* = 33).

Variable	Subgroup	Number of studies	Sample size	*I* ^2^	ES (95% CI)	*p* value
Sample size^∗^	≥822	9	15,379	99.57%	34.36 (24.76, 43.96)	<0.001
	<822	24	11,749	99.54%	38.56 (27.71, 49.41)	<0.001
Publication year	2010-2015	9	13,952	99.52%	34.09 (24.46, 43.72)	<0.001
	2016-2020	24	13,176	99.58%	38.66, (28.41, 48.91)	<0.001
Study setting	Community-based	21	19,801	99.51%	40.97 (33.38, 48.55)	<0.001
	Facility-based	12	7327	99.56%	31.2 (19.4, 42.99)	<0.001

Note: ^∗^sample size for subgroup analysis categorized by taking the mean sample size.

## Data Availability

All relevant datasets used and/or analyzed are available upon reasonable request to the corresponding author.

## Supplementary Materials

Supplementary Materials Additional file 1: Preferred Reporting Items for Systematic Reviews and Meta-Analyses: the PRISMA statement 2009 checklist.
