# Supplementary material for: Postpartum Family Planning Use and Its Determinants among Women of the Reproductive Age Group in Low-Income Countries of Sub-Saharan Africa: A Systematic Review and Meta-Analysis
Source: Int J Reprod Med. 2021 Aug 20;2021:5580490. doi: 10.1155/2021/5580490 (PMC8403053; doi:10.1155/2021/5580490)
Supplement: Supplementary Materials — Additional file 1: Preferred Reporting Items for Systematic Reviews and Meta-Analyses: the PRISMA statement 2009 checklist. [file 5580490.f1.docx]

**PRISMA Flow Diagram**

Identification

Additional records identified through other sources (n = 8)

Articles identified through database searching (n = 735)

Articles after duplicates removed
(n = 609)

Screening

Articles excluded by their tittles (n=320)

Articles excluded by their abstract (n =241 )

Articles screened based on titles and abstract (n = 609)
(n = )

Full-text articles excluded, with reasons (n = 15)

- Outcome of interest not reported (n=3)
- Conducted out of setting (n=2)
- Due to Outcome measurement (n=4)
- Due to study population (n=2)
- Report only LARC (n=3)
- No Data on factors/determinants (n=1)

Eligibility

Full-text articles assessed for eligibility (n = 48)

Studies included in qualitative synthesis (n = 33)

Included

Studies included in quantitative synthesis (meta-analysis) (n = 33)

**Figure 1: Description of schematic presentation of the PRISMA flow diagram to select and include studies, 2020.**
